# Preoperative Near-Infrared (NIR) Vein Visualization in Zygomatic Implant Perforated (ZIP) Flap †

**DOI:** 10.3390/cmtr19020019

**Published:** 2026-04-01

**Authors:** Yoram Fleissig, Jhonatan Elia, Nir Hirshoren, Amalia Sabato, Eleonora Ginzburg, Jawad Abu Tair, Jeffrey M. Weinberger, Shay Sharon

**Affiliations:** 1 Department of Oral and Maxillofacial Surgery, Hadassah Medical Center, Faculty of Dental Medicine, Hebrew University of Jerusalem, P.O. Box 12272, Jerusalem 9112102, Israel; 2 Department of Plastic Surgery, Hadassah Medical Center, Faculty of Medicine, Hebrew University of Jerusalem, P.O. Box 12272, Jerusalem 9112102, Israel; 3 Department of Otolaryngology—Head and Neck Surgery, Hadassah Medical Center, Faculty of Medicine, Hebrew University of Jerusalem, P.O. Box 12272, Jerusalem 9112102, Israel; 4 Maxillofacial Rehabilitation Unit, Hadassah Medical Center, Faculty of Dental Medicine, Hebrew University of Jerusalem, P.O. Box 12272, Jerusalem 9112102, Israel

**Keywords:** zygomatic implant perforated flap, near-infrared vein visualization, maxillectomy reconstruction

## Abstract

Zygomatic implant perforated (ZIP) flap reconstruction offers immediate surgical rehabilitation following maxillectomy, integrating oncologic zygomatic implants with a fascio-cutaneous free flap. A critical technical challenge is safely perforating the free flap skin paddle to accommodate implants’ abutments without damaging its vasculature. Near-infrared (NIR) vein visualization technology provides real-time mapping of subcutaneous vessels and has been widely investigated in settings such as pediatric intravenous (IV) cannulation. By projecting vein pathways onto the skin, NIR visualization facilitates precise vascular identification, potentially reducing complications. We describe a case of ZIP flap reconstruction in a 25-year-old patient utilizing NIR vein visualization to preemptively locate flap vasculature and minimize the risk of vessel puncture. Our discussion places these findings within the context of the existing literature on NIR devices, underscoring their benefits of non-invasive operation, rapid imaging, and minimal need for advanced operator skills, and highlighting their utility in microvascular reconstructive surgery.

## 1. Introduction

Surgical reconstruction following maxillectomy is both complex and challenging, with ongoing debate regarding the optimal reconstruction approach. Each method has distinct advantages and disadvantages [1]. While obturators can provide satisfactory outcomes, issues such as nasal leakage, retention, and resistance often arise. In certain cases, reconstruction using composite microvascular free tissue transfer is preferred, yielding excellent results. However, it is estimated that fewer than 50% of patients achieve dental rehabilitation following maxillary free flap reconstruction due to various challenges [2]. Even when successful, composite free flap reconstructions often delay dental rehabilitation by 12 to 18 months [3]. While the concept of a maxillary jaw in a day offers a promising solution, it is not extensively reported in the literature, and concerns about higher flap failure rates were raised, especially in malignant cases [2]. Edentulism has a well-documented negative impact on patients’ quality of life. Achieving acceptable functional and aesthetic outcomes through microvascular reconstruction, dental implants, and prostheses can significantly enhance patient well-being [4].

Rogers and Butterworth introduced the zygomatic implant perforated (ZIP) flap technique, a method for immediate surgical reconstruction and rapid postoperative prosthodontic rehabilitation with a fixed dental prosthesis following low-level maxillectomy [5]. Although Hirsch et al. previously described a similar approach, their method involved placing the zygomatic implants at a second stage [6]. In the ZIP flap technique, zygomatic oncologic implants are inserted immediately after maxillary tumor ablation, while a second surgical team simultaneously harvests a fascio-cutaneous free flap, typically from the radial forearm.

The free flap is meticulously perforated to accommodate the zygomatic implant abutments before being anastomosed. Recent outcomes from a series of 35 cases highlight the potential of this technique, demonstrating its predictability, rapid return to function, and high levels of patient satisfaction [7].

Although this technique is relatively straightforward, microvascular surgeons may find the perforation of the free flap skin paddle challenging, particularly when the flap has not yet been anastomosed, and the superficial vasculature is inadequately visible.

Near-infrared (NIR) vein visualization technology allows for the real-time mapping of subcutaneous vasculature directly onto the skin’s surface. NIR light penetrates the skin and subcutaneous fat, where it is absorbed by blood flowing through the veins, making the veins appear dark against the lighter background of the surrounding tissues. NIR vein visualization technology is widely used in modern hospitals, particularly in emergency medicine and pediatric departments, as well as in anesthesiology, intensive care, and oncology units.

This paper aims to describe the utility of near-infrared (NIR) vein visualization in preventing vessel puncture during flap skin paddle perforation.

## 2. Case Presentation

**Figure 1:**
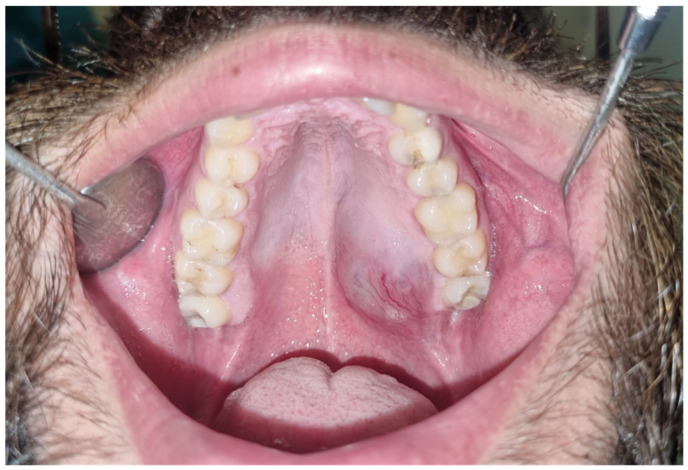
A 25-year-old male with a two-year history of a growing mass in the left palate.

**Figure 2:**
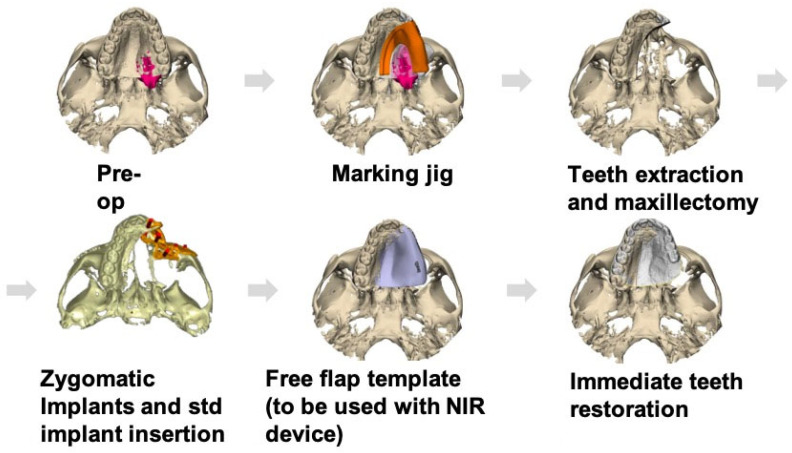
CAD-CAM workflow planning, marking jig, low-level maxillectomy, placement of two oncologic zygomatic implants and a standard long implant at the upper left second incisor site, free-flap template to transfer perforation sites to the skin paddle in conjunction with vasculature mapping using NIR vein visualization, and plan for immediate teeth restoration.

A 25-year-old male presented to our outpatient clinic with a two-year history of a growing mass in his left palate (Figure 1). An incisional biopsy confirmed a diagnosis of low-grade mucoepidermoid carcinoma. Oncologic workup included CT, MRI, CBCT, and dental scans. The CAD-CAM workflow (Figure 2) involved using ProPlan CMF software (Materialise, Leuven, Belgium) to plan for a marking jig for performing the planned resection with adequate margins and low-level maxillectomy. The EZplan Real Guide planning software (Noris Medical zygomatic implants, Noris Medical, Nesher, Israel) was used to plan the placement of two oncologic zygomatic implants (50 mm and 37.5 mm long), and a standard 16 mm long implant at the upper left second incisor site, which was sacrificed to enhance dental prosthesis stability. A patient-specific titanium surgical guide was fabricated to ensure precise implant placement, and the dental prosthesis was designed using Meshmixer ver. 3.5 (Autodesk Inc., San Francisco, CA, USA) software and manufactured by a 3D printer (Formlabs, Somerville, MA, USA). To mark preferred skin paddle perforation sites for zygomatic implants’ abutments, we used a free-flap 3D printed elastic template that could transfer the specific location.

**Figure 3:**
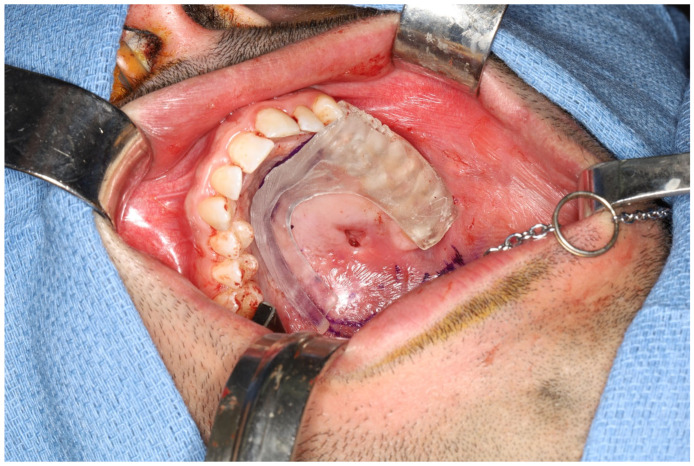
Marking jig used to mark the specific soft tissue resection border.

**Figure 4:**
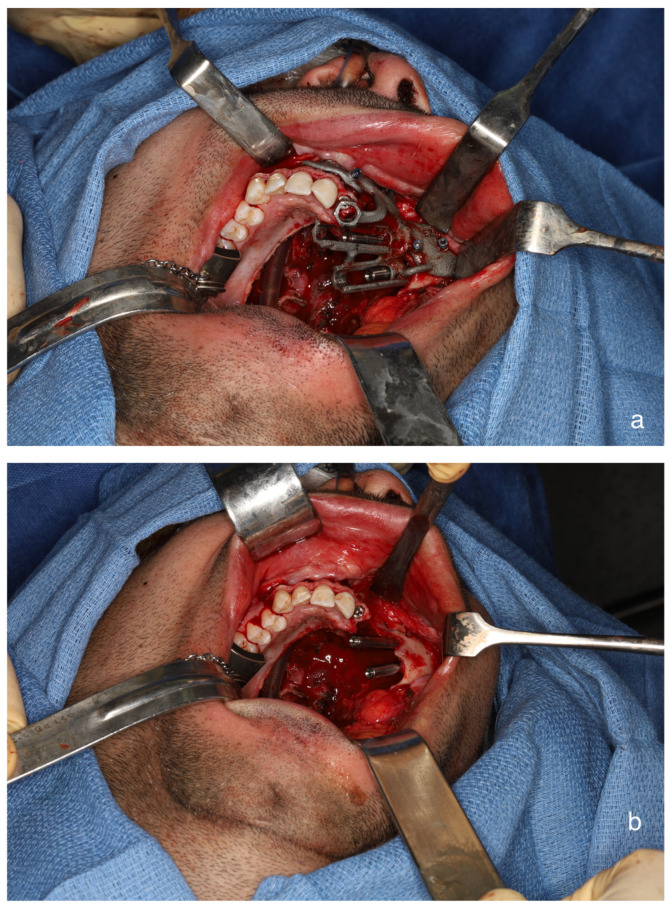
(**a**) Surgical guide for zygomatic implants and standard implant, fits well to post-resection maxillary and zygomatic defect boundaries. (**b**) Inserted dental implant and two zygomatic implants, all achieving excellent primary stability.

**Figure 5:**
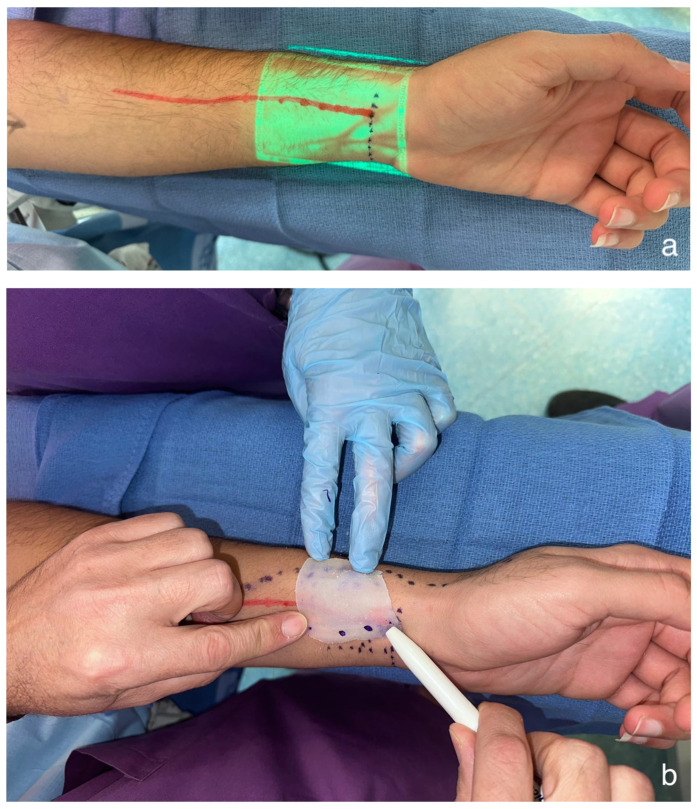

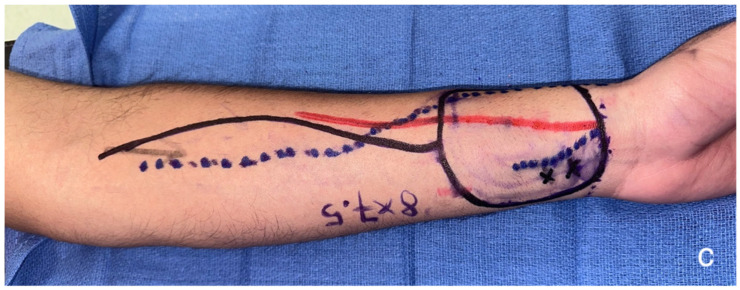
(**a**) Near-infrared vein visualization mapping the superficial vasculature of the radial forearm. Red line marks represent the radial artery. (**b**) A free-flap template was used to verify and transfer the preferred perforation sites (dot marks) for the zygomatic implants’ abutments. (**c**) Radial forearm free flap markings (black marker). Red line marks the radial artery, blue dotted line marks the cephalic vein, and black asterisks mark the planned flap perforation for zygomatic implants, selected in a vascular-safe area.

**Figure 6:**
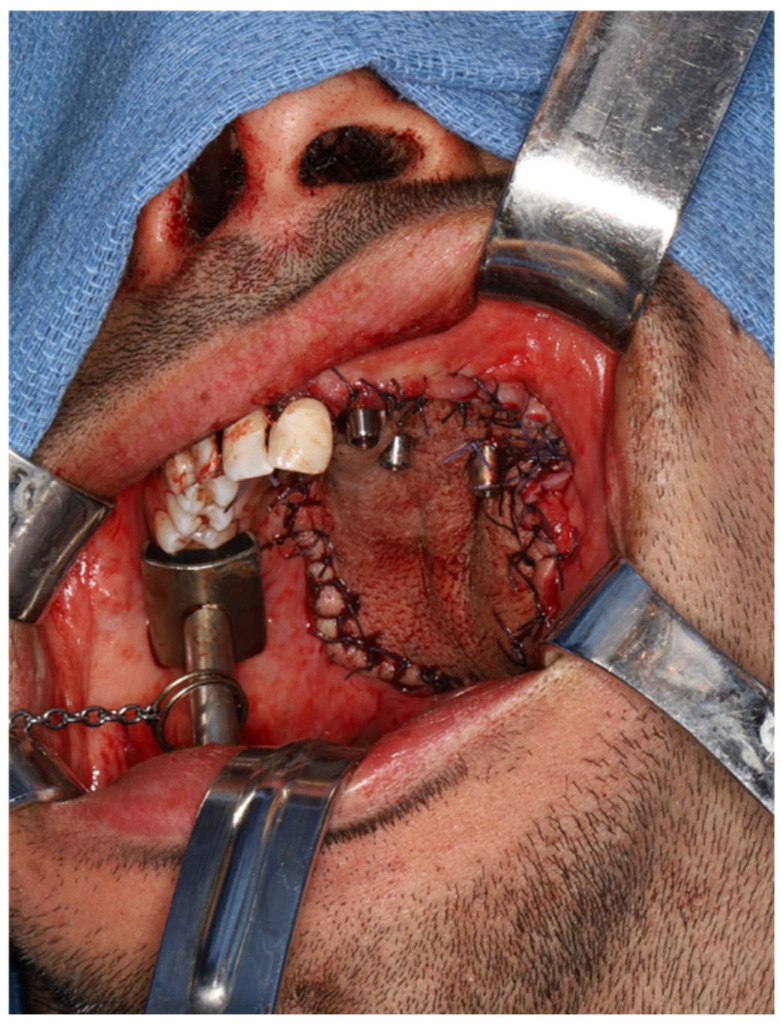
Insetting of radial forearm free flap into maxillectomy defect and perforating two zygomatic implants and one standard dental implant.

**Figure 7:**
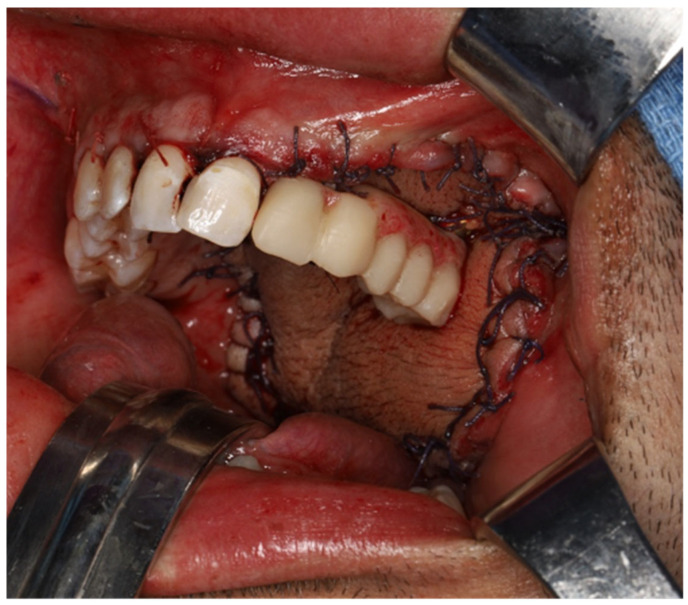
Immediate rehabilitation with a prefabricated dental prosthesis that was fixed to the implants using a multi-unit system.

**Figure 8:**
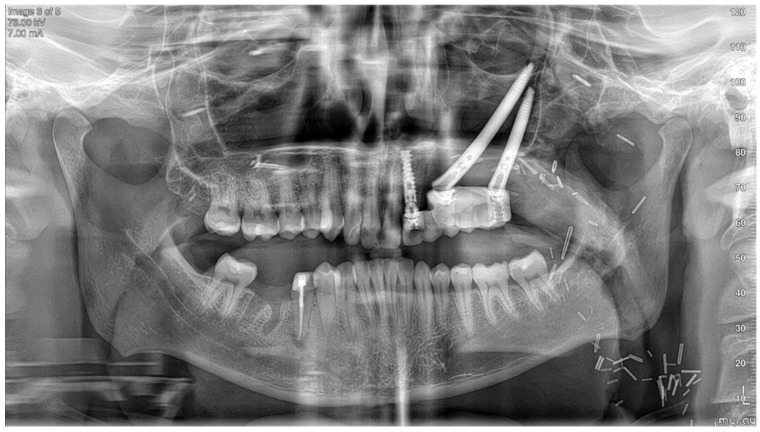
Postoperative panoramic X-ray.

The surgical procedure included a tracheostomy, elective selective neck dissection at level Ib, and vessel preparation. A low-level hemi-maxillectomy (Brown IIB) was performed through an intraoral approach, extending over the midline and including part of the soft palate and through the upper left canine socket. The marking jig was used to delineate the specific soft tissue resection border (Figure 3). The upper left second incisor was extracted, and the surgical guide was used to place the dental implant and two zygomatic implants, all achieving excellent primary stability (Figure 4). Before harvesting the radial forearm free flap, near-infrared vein visualization was utilized to map the superficial vein network and perforators from the radial artery, marking vascular-free area and optimal perforation locations for the zygomatic implants’ abutments. A CAD-CAM free-flap template was used to verify and transfer the preferred perforation sites for the zygomatic implants’ abutments (Figure 5a,b). In this case, according to the surgical resection site, expected emergence of the implants, flap geometry and the chosen direction of the vascular pedicle (posteriorly), perforations were planned at the medial side of the radial forearm free flap, far from the radial artery, but adjacent to the entangled superficial vein network (Figure 5c). The vascular pedicle was tunneled to the left neck, and flap insetting was completed with perforations at the pre-marked sites. Before perforating the skin paddle, the zygomatic implants’ abutments were palpated, and we verified that their actual placement fits the plan and skin markings. Perforation was done meticulously using blade #11 and fine Mosquito forceps (Figure 6). After vascular anastomosis, the prefabricated dental prosthesis was fixed to the implants using a multi-unit system (Figure 7). Final pathology report described intermediate to high-grade mucoepidermoid carcinoma with perineural invasion and free surgical margins. Postoperative panoramic x-ray presents the implant location and temporary restoration (Figure 8).

## 3. Technique

To facilitate reproducibility, the CAD-CAM workflow is described step by step (Figure 2).

**Figure 9:**
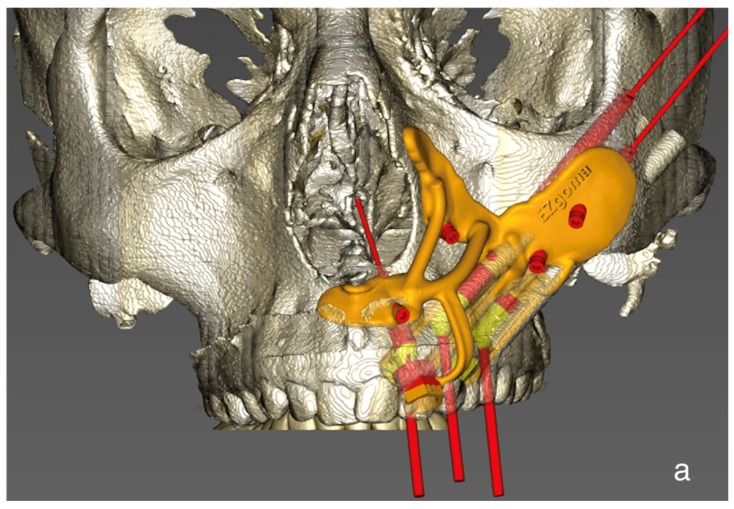

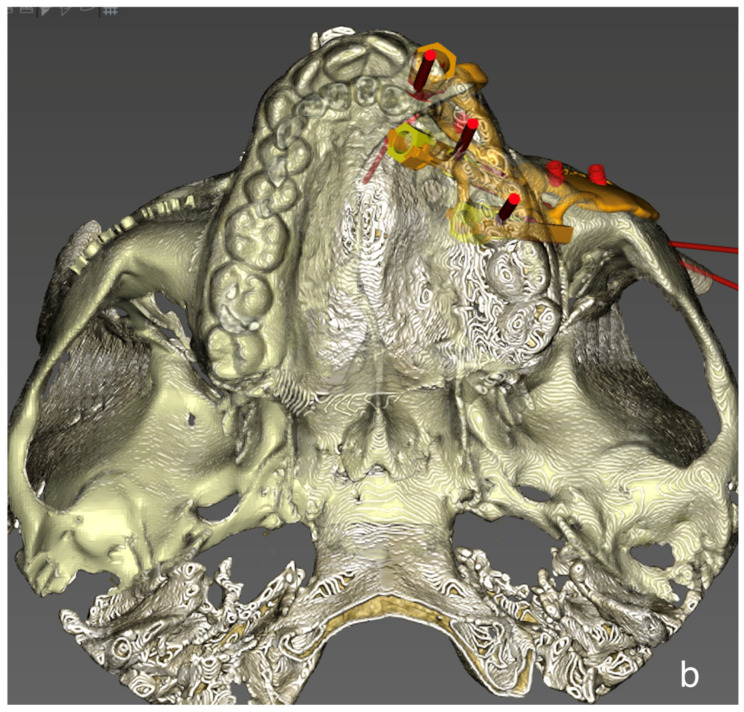
(**a**) Plan for surgical guide for zygomatic implants and standard implants—frontal view, demonstrating angulated and parallel abutments. (**b**) Plan for surgical guide for zygomatic implants and standard implants—inferior view, demonstrating abutment angles coinciding with the dental arch. A thin red line indicates the long axis of the implant.

**Figure 10:**
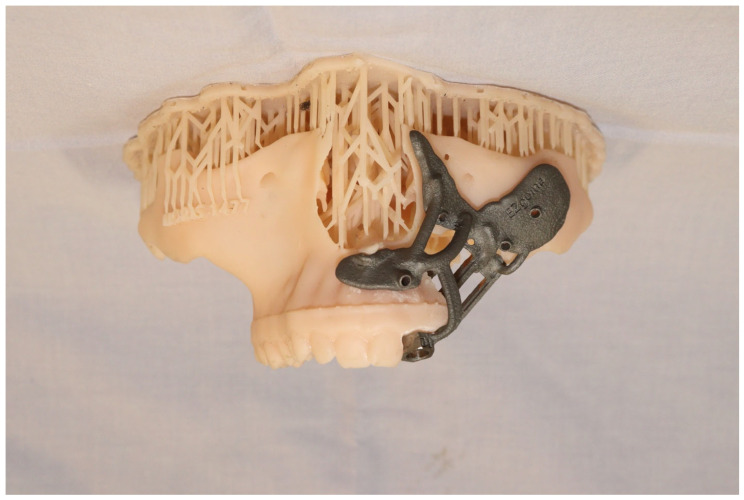
Printed 3D model to assist the surgeon in understanding what surgical defect to expect and how to fit the surgical guide.

**Figure 11:**
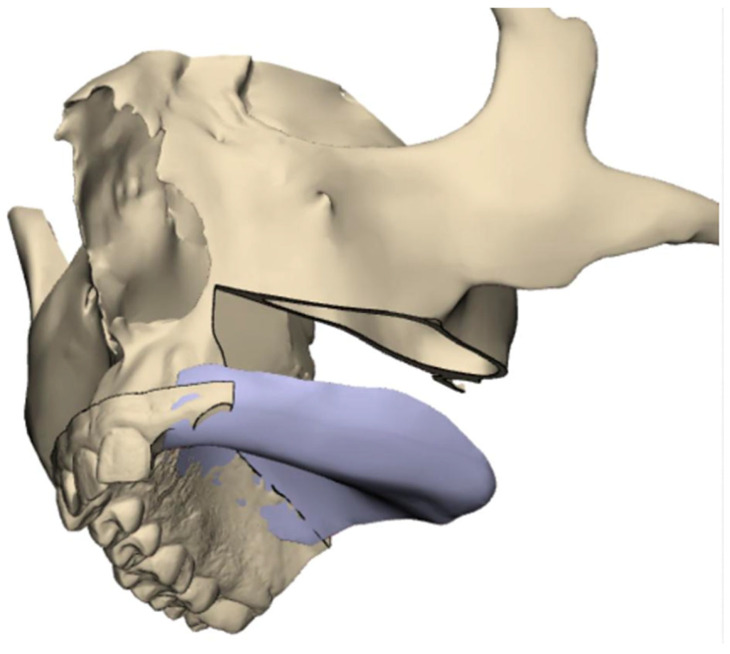
Plan for a free-flap template (printed from elastic material) to measure precisely for free flap size and geometry, and for transferring the preferred sites of perforation to the skin paddle.

1. Tumor marking and resection planning: Using CAD-CAM, mark the tumor and plan sufficient oncologic resection margins using a marking jig.
2. Virtual resection: Execute virtual resection according to the tumor size and planned margins.
3. Implant planning: Using a dental scan of both jaws, plan the positions of zygomatic implants and abutment emergence to fit the dental arch and occlusion. As a guideline, we aim for 15 mm vertical distance between the implant platform and opposing teeth.
4. Surgical guide preparation: Plan a surgical guide for both zygomatic and standard implants (Figure 9). Ensure that the guide fits accurately to the post-resection maxillary and zygomatic defect boundaries. We recommend printing a 3D model to help the surgeon visualize the expected surgical defect (Figure 10). For cases with high uncertainty, surgical cutting guides can be printed (though they were not used in this case).
5. Free flap template planning: Print a free-flap template from elastic material to precisely measure flap size and geometry (Figure 11). After determining the side of vascular pedicle emergence, mark the preferred perforation sites for zygomatic implants on the template.
6. Vascular mapping: Using an NIR vein visualization device, mark the superficial vasculature on the skin paddle. Place the free flap template over the skin paddle, aligning the preferred perforation sites with a vascular-safe area.
7. Pre-perforation verification: After free flap harvesting and insetting, palpate the abutments over the zygomatic implant just before perforating the flap to ensure proper alignment of the planned perforation site.
8. Flap Perforation: Create a stab incision with a #11 blade and gently dilate with fine Mosquito forceps. Avoid over-enlarging the perforation, maintaining soft tissue tension to allow abutment insertion while preventing outward displacement. Use sutures as needed to reinforce tissue tension around the abutment.
9. Immediate restoration: Following free flap anastomosis, the prosthodontic team can proceed with immediate dental restoration. This restoration can be planned using CAD-CAM, based on the planned zygomatic implants, abutment emergence, and the patient’s occlusion.

## 4. Discussion

The NIR vein visualization device has been previously employed in reconstructive procedures, such as the design of a venous flap [8]. Its ability to enhance precision in identifying vascular structures makes it an invaluable tool in both routine and specialized medical applications. Much of the existing literature on NIR vein visualization focuses on intravenous (IV) cannulation, particularly in pediatric patients, where the aim is to shorten cannulation time, reduce the number of attempts, and minimize pain or anxiety [9,10,11]. Although many of these studies show mixed or inconclusive results, likely due to population heterogeneity and varying operator skill, some also suggest potential benefits for individuals with difficult venous access, such as reduced complications like hematomas [12,13]. Crucially, the core principle remains the same: direct visualization of otherwise obscured veins can expedite procedures and lower the risk of vascular injury.

These insights align with our use of NIR vein visualization technology in flap surgery. In the pediatric realm, the key advantages include non-contact operation, real-time imaging, ease of learning, and no requirement for injected agents [9,14]. The same features can be highly beneficial when performing delicate procedures such as perforating a free flap skin paddle. By providing a clear visualization of the superficial vasculature, NIR vein visualization devices help avoid accidental vessel puncture, minimize bleeding, and potentially shorten operative time. The device is simple to use, typically with a working distance of 20–30 cm. It does not require a tourniquet, and no special setting is necessary to gain a good image quality.

Recent studies in adult and elderly populations [13] have further underlined the role of NIR vein visualization devices in reducing complications and patient distress. While some randomized trials found no significant improvement in first-attempt success rates or procedure duration, these devices were nonetheless associated with decreased hematoma formation and lower anxiety or depression scores, pointing toward improved overall patient comfort [13]. Although these findings stem from IV cannulation contexts, the underlying principle of careful vascular visualization can be readily translated to surgical procedures like ZIP flap reconstruction.

The radial forearm free flap is commonly the preferred choice for ZIP flap reconstruction due to its favorable thickness and pliability. Its superficial vasculature consists of the cephalic and basilic veins, which are typically visible to the naked eye. However, smaller intercommunicating veins on the volar side, as well as radial artery perforators, may be overlooked due to their random distribution and size. After harvesting the radial forearm flap, the veins become depleted and are no longer visible, making NIR vein visualization particularly useful preoperatively. This straightforward, non-invasive technique is time-efficient and can prevent vascular puncture and the complications of uncontrolled bleeding, especially when the flap is already sutured in place within the oral cavity. The device is relatively inexpensive and widely available in hospital settings. Although never discussed, we believe that perforating the free flap skin paddle is a challenging step. Incorporating the NIR vein visualization method could help address the concerns of reconstructive surgeons, particularly during their initial cases, and reduce the likelihood of abandoning this effective reconstruction option.

The practical advantages of NIR vein visualization devices, like non-invasiveness, ease of use, and immediate visualization, make them well-suited for incorporation into ZIP flap workflows. Although more comparative research is warranted, our experience suggests that the principles underlying the NIR vein visualization device’s success in venous cannulation can indeed inform and improve surgical flap handling.

This case is notable for being performed on a young patient (25 years old), whereas most reported cases involve individuals in their seventh decade of life [7]. Additionally, ZIP flap reconstructions are typically bilateral, involving quad zygomatic implants. These two factors raise concerns about the long-term success and durability of this case. We also highlight the utility of CAD-CAM technology in the ZIP flap procedure, a concept previously suggested [15]. It is important to note in this context that the use of NIR vein visualization technology for skin-paddle perforation in free flap is independent of the CAD-CAM workflow and can be applied without it. However, since zygomatic implant placement requires a high level of technical skill, the use of CAD-CAM and surgical guides can be an excellent option for young surgeons. Although this approach demands significant time and resources preoperatively, it simplifies the execution of initial cases and enhances surgical precision.

## 5. Conclusions

NIR vein visualization can significantly aid in the perforation of a radial forearm free flap during zygomatic implant perforated (ZIP) flap reconstruction. By clearly delineating superficial vasculature in real time, it helps avoid vessel injury and reduces the risk of complications, which can be especially advantageous for surgeons undertaking this procedure for the first time. Although the literature on NIR devices spans diverse clinical contexts, particularly pediatric venous cannulation, its core benefits of non-invasive operation, ease of use, and rapid vascular mapping are directly applicable to complex reconstructive surgery. Incorporating NIR vein visualization into ZIP flap workflows can streamline the surgical process, contribute to flap integrity, and potentially accelerate patient recovery.

## Data Availability

No new data were created or analyzed in this study.
